# Functional enrichment analysis based on long noncoding RNA associations

**DOI:** 10.1186/s12918-018-0571-0

**Published:** 2018-04-24

**Authors:** Kuo-Sheng Hung, Chung-Chi Hsiao, Tun-Wen Pai, Chin-Hwa Hu, Wen-Shyong Tzou, Wen-Der Wang, Yet-Ran Chen

**Affiliations:** 1 Department of Computer Science and Engineering, National Taiwan Ocean University, Keelung, Taiwan; 2 Department of Bioscience and Biotechnology, National Taiwan Ocean University, Keelung, Taiwan; 3 Department of Bioagricultural Science, National Chiayi University, Chiayi, Taiwan; 4 Agricultural Biotechnology Research Center, Academia Sinica, Taipei, Taiwan; 5 Center of Excellence for the Oceans, National Taiwan Ocean University, Keelung, Taiwan

**Keywords:** lncRNA, Differential expression, Gene ontology (GO), KEGG, Neuron development

## Abstract

**Background:**

Differential gene expression analysis using RNA-seq data is a popular approach for discovering specific regulation mechanisms under certain environmental settings. Both gene ontology (GO) and KEGG pathway enrichment analysis are major processes for investigating gene groups that participate in common biological responses or possess related functions. However, traditional approaches based on differentially expressed genes only detect a few significant GO terms and pathways, which are frequently insufficient to explain all-inclusive gene regulation mechanisms.

**Methods:**

Transcriptomes of survivin (*birc5*) gene knock-down experimental and wild-type control zebrafish embryos were sequenced and assembled, and a differential expression (DE) gene list was obtained for traditional functional enrichment analysis. In addition to including DE genes with significant fold-change levels, we considered additional associated genes near or overlapped with differentially expressed long noncoding RNAs (DE lncRNAs), which may directly or indirectly activate or inhibit target genes and play important roles in regulation networks. Both the original DE gene list and the additional DE lncRNA-associated genes were combined to perform a comprehensive overrepresentation analysis.

**Results:**

In this study, a total of 638 DE genes and 616 DE lncRNA-associated genes (lncGenes) were leveraged simultaneously in searching for significant GO terms and KEGG pathways. Compared to the traditional approach of only using a differential expression gene list, the proposed method of employing DE lncRNA-associated genes identified several additional important GO terms and KEGG pathways. In GO enrichment analysis, 60% more GO terms were obtained, and several neuron development functional terms were retrieved as complete annotations. We also observed that additional important pathways such as the FoxO and MAPK signaling pathways were retrieved, which were shown in previous reports to play important roles in apoptosis and neuron development functions regulated by the survivin gene.

**Conclusions:**

We demonstrated that incorporating genes near or overlapped with DE lncRNAs into the DE gene list outperformed the traditional enrichment analysis method for effective biological functional interpretations. These hidden interactions between lncRNAs and target genes could facilitate more comprehensive analyses.

## Background

Embryonic development of the nervous system mainly relies on complex interactions between extrinsic signaling factors and intrinsic regulation of gene expression. Different environmental conditions, such as temperature, diet, toxin levels, and chemical levels can change the gene expression profiles from their usual patterns and lead to defects in neuron development. Recent research has indicated that hypoxic (~ 1% O_2_) environments also affect neuron-related gene expression [1]. Evidence has shown that upregulated expression of hypoxia-inducible factor (HIF) in response to the absence of oxygen leads to a series of downregulated gene expressions and induces physiological and metabolic changes in neuron development [2].

Our previous studies showed that zebrafish under hypoxic embryogenesis conditions exhibited upregulated expression of *hif2α*, which is involved in neural differentiation processes via expression regulation of the survivin family (*birc5* gene) [3]. Survivin is a protein containing a BIR (Baculovirus Inhibitor of apoptosis Repeat) domain and an α-helical coiled-coil domain that is expressed in neural precursor cells and neural progenitor cells during development. Survivin, as an IAP (inhibitor of apoptosis proteins) family member that binds to caspases, can inhibit apoptosis [4, 5]. Further investigation into the role of survivin-related genes in neuron development is important to the understanding of the basic mechanisms controlling neural cell growth and signal transduction pathways.

Noncoding RNAs (ncRNAs) are abundant in cells, are not translated into proteins, and are functionally categorized into two major types. The first type is structural noncoding RNA, which includes transfer RNAs, ribosomal RNAs, small nucleolar RNAs, and small nuclear RNAs; the second type is regulatory noncoding RNA, which includes microRNAs, small interfering RNAs, extracellular RNAs, piwi-interacting RNAs, and long noncoding RNAs, among others. Several studies have shown that microRNAs (miRNAs) and long noncoding RNAs (lncRNAs) possess gene expression regulation properties in neuronal system development [3, 6, 7]. The regulatory relationships between miRNA and genes have already attracted attention and have been verified by satisfactory experiments [8]. Comparatively few lncRNAs have been studied in animal models, and a comprehensive functional analysis of most lncRNAs remains challenging because lncRNAs are not evolutionarily conserved among related species.

Several kinds of lncRNAs are categorized based on their genomic loci. Genomic locations of lncRNAs associated with related protein-coding genes can be grouped as sense or antisense transcript overlapping and as promoter-associated, intronic, or intergenic. A promoter-associated lncRNA is transcribed from the upstream region of the protein-coding genes [9], an intronic lncRNA possesses a transcribed region overlapped with the intron segments of protein-coding genes, and an intergenic lncRNA is located between two protein-coding genes [10]. Additionally, some special lncRNAs possess coding ability and form as regular peptides, which probably perform essential functions [11, 12]. Many lncRNAs were systematically identified by Pauli et al. through a time-series of RNA-seq experiments related to zebrafish embryo development. The results demonstrated that lncRNAs are expressed significantly at early embryonic stages and possess stage-specific and tissue-specific expression characteristics [13].

One traditional approach for understanding regulation mechanisms is analyzing differentially expressed genes (DEGs) through Gene Ontology (GO) terms and pathway (such as KEGG pathways) enrichment analysis using hypergeometric distributions. Unfortunately, sometimes only a few significant GO terms and pathways can be identified using the limited number of DEGs, which leads to difficulty in explaining all-inclusive gene regulation mechanisms.

Normally, the regulation of ncRNA expression is not considered to be within the overall RNA regulation network. However, many genes that undergo no significant changes in expression are regulated mainly through induction or inhibition by ncRNAs; thus, discounting the regulatory function of ncRNAs may lead to inadequate identification of significant GO terms or functional pathways. An alternative solution is to incorporate lncRNAs in regulation analysis and to apply co-expression network methods for a comprehensive analysis by using tools such as ncFANs [14], lncRNAtor [15], and Co-LncRNA [16]. Although co-expression network analysis provides possible data to bridge gaps in functional annotations, it has some limitations. Serin et al. indicated that large data connections would make co-expression networks complicated and explaining biological functionality difficult. Additionally, the connection between genes in a co-expression network does not necessarily imply the existence of a regulatory relationship in biological systems [17].

In this study, we proposed a method of combining genes near or overlapped with differentially expressed lncRNAs (DE lncRNAs) and DEG lists for functional enrichment analysis. We expect that after incorporating these genes, functional analysis will provide more significant GO terms and KEGG pathway annotations than the traditional approach of adopting DEGs only. To validate our hypothesis, a knock-down experiment on *birc5a* with a morpholino (Birc5aMO) in zebrafish embryo neuron development and an RNA-seq experiment for detecting DEGs and DE lncRNAs were performed. The detailed procedures and results are introduced and discussed in the following sections.

## Methods

### RNA-Seq

**Fig. 1:**
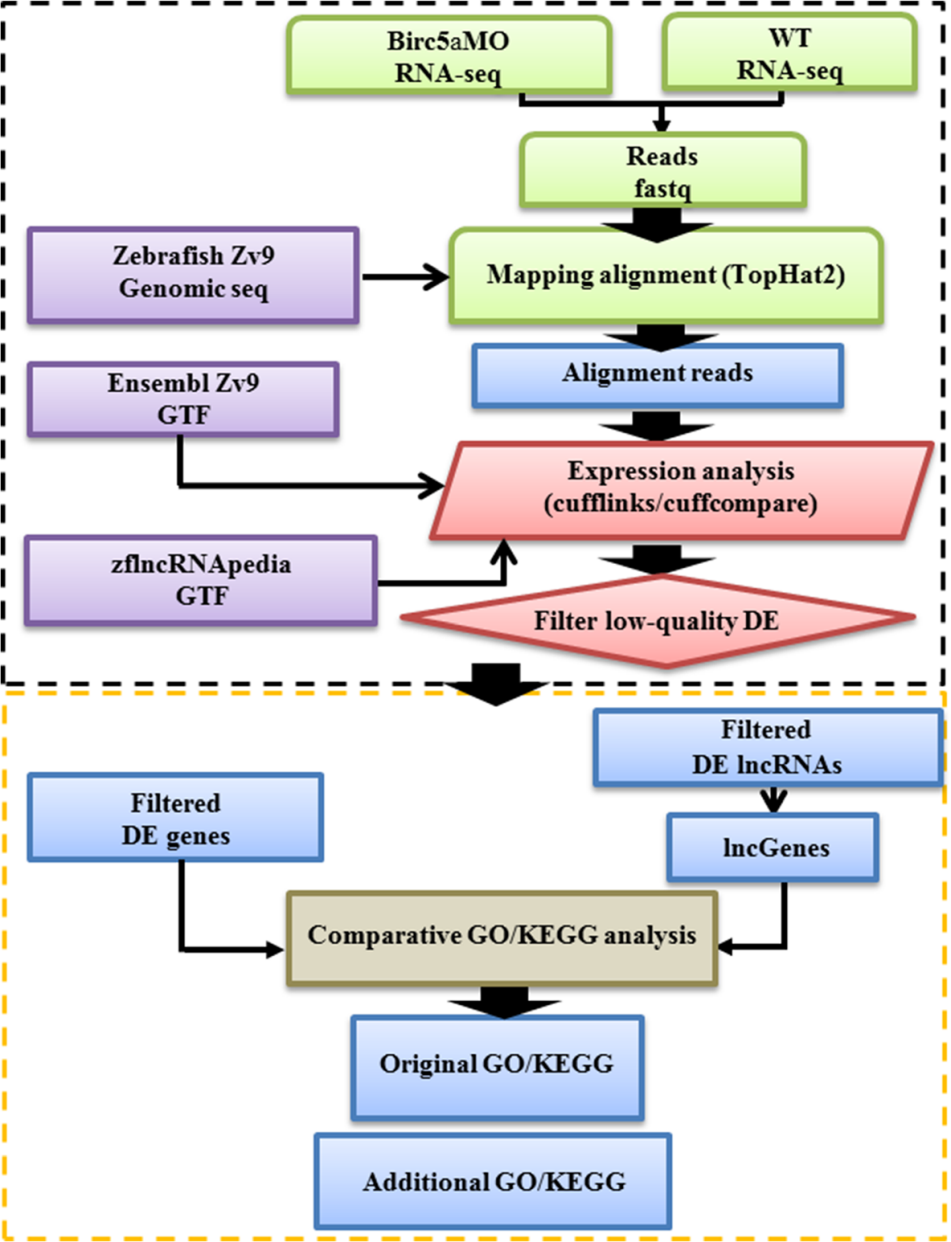
RNA-seq analysis flowchart. Black dotted box: paired-end sequence reads from Birc5aMO and WT RNA sequencing aligned by TopHat2, and expression changes obtained by cufflinks and cuffcompare. Yellow dotted box: GO and KEGG pathway enrichment analysis by using DE genes (traditional) and adding lncGenes for identifying additional significant annotations

The proposed system configuration is shown in Fig. 1. First, *birc5a* morpholino-treated (Birc5aMO) and untreated wild type (WT) zebrafish embryos were collected 20 h post-fertilization (hpf), and the total RNA was extracted from each sample (with two repeats) using TRIzol reagent. After RNA preparation, the cDNA libraries were constructed by Genomics (NTC, Taiwan). The cDNA libraries were generated and sequenced with a NextSeq 500 sequencer (Illumina, CA, USA) with paired-end short read fragments 100 bp in length. The resulting fastq sequence files were further filtered and trimmed to remove contaminating artifacts and low-quality base calls for the following sequence mapping alignment.

### Short Read Alignment and Expression Calculation.

Filtered reads were aligned to a zebrafish genome sequence (Ensembl release 79 version Zv9) by TopHat2 [18]. The mapped reads from bam files were further analyzed and normalized to corresponding expression patterns and values by Cufflinks [19]. The unit of expression value is FPKM (Fragments Per Kilobase of transcript per Million). Gene expression annotation was analyzed with the Ensembl Zv9 transcript GTF file. For lncRNA analysis, the GTF file from zflncRNApedia [20] was applied for unified annotation of lncRNAs in zebrafish. To compare differential expression (DE) of genes and lncRNAs between the Birc5aMO and WT control datasets, Cuffcompare [19] was applied by using the average FPKM of repeat samples. Fold changes in gene and lncRNA expression of > 1.5 or < − 1.5 were considered to indicate significantly expressed genes in enrichment analysis. Genes nearby or overlapped with DE lncRNAs (within a default parameter of 5 kb upstream or downstream of protein-coding regions) were identified and considered to be DE lncRNA-associated genes (lncGenes).

### GO and KEGG pathway enrichment analysis.

The annotated DE genes and lncGenes were further explored for their corresponding biological meaning by GO annotations retrieved from the Gene Ontology Consortium (version 2017.03.01) [21] and mapped against biological pathway information in the KEGG database (version 0.7.2).

For GO and KEGG pathway enrichment analysis, we applied hypergeometric distribution statistical theory to calculate *p*-values for each pathway and each GO term. The formula for traditional over-representation analysis is represented in Eq. (1).

1 $$ {P}_j\left( at\  least\ i\  genes\right|N,n,K\Big)=\sum \limits_{\boldsymbol{k}=\boldsymbol{i}}^{\mathbf{\min}\left(\boldsymbol{n},\mathbf{K}\right)}\frac{\left(\genfrac{}{}{0pt}{}{\boldsymbol{K}}{\boldsymbol{k}}\right)\left(\genfrac{}{}{0pt}{}{\boldsymbol{N}-\boldsymbol{K}}{\boldsymbol{n}-\boldsymbol{k}}\right)}{\left(\genfrac{}{}{0pt}{}{\boldsymbol{N}}{\boldsymbol{n}}\right)} $$

where *N* is the number of all unique genes mapped against the reference dataset, *n* is the number of DE genes, *K* is the number of all genes annotated in a specific GO term or a specific KEGG pathway, and *k* is the number of DE genes mapped to the specific GO term or the specific KEGG pathway. The *p*-value for the *j*^th^ GO term or the *j*^th^ KEGG pathway is denoted by *P*_*j*_. The proposed novel GO and KEGG pathway enrichment analysis considers the additionally identified DE lncRNAs and their associated genes (lncGenes). Therefore, the modified formula for over-representation analysis is represented in Eq. (2).

2 $$ {P}_j\left( at\ l\mathrm{e} as\mathrm{t}\ i\  genes\right|N,n,K,r\Big)=\sum \limits_{\boldsymbol{k}=\boldsymbol{i}}^{\mathbf{\min}\left(\boldsymbol{n}+\boldsymbol{r},\boldsymbol{K}\right)}\frac{\left(\genfrac{}{}{0pt}{}{\boldsymbol{K}}{\boldsymbol{k}+\boldsymbol{r}}\right)\left(\genfrac{}{}{0pt}{}{\boldsymbol{N}-\boldsymbol{K}}{\left(\boldsymbol{n}+\boldsymbol{r}\right)-\left(\boldsymbol{k}+\boldsymbol{r}\right)}\right)}{\left(\genfrac{}{}{0pt}{}{\boldsymbol{N}}{\boldsymbol{n}+\boldsymbol{r}}\right)} $$

where *r* is the number of lncGenes and all other parameters (*N*, *n*, *K*, *k*, and *P*_*j*_) denote the same values as in Eq. (1).

When *P*_*j*_ is less than or equal to 0.05, the specified GO term or KEGG pathway was considered a significant functional annotation (*p-*value <= 0.05). In the GO structure, the identified significant parent GO terms with significant child terms were removed according to the GO tree structure relationship for the most specific and accurate annotations.

## Results

After performing read quality check procedures on the RNA-seq datasets, a total of 20,759,640 (Birc5aMO repeat 1), 21,003,970 (Birc5aMO repeat 2), 22,221,792 (WT repeat 1), and 22,456,290 (WT repeat 2) reads were retained as high-quality reads for functional analysis. TopHat2 and Cufflinks were used for sequence mapping and differential expression analysis of the Birc5aMO and WT control datasets; two resulting gene sets, including 638 DE genes and 438 DE lncRNAs, were identified with significant fold changes relative to 29,806 unigenes and 17,488 lncRNAs, respectively, based on the FPKM normalization mechanism (see Table 1).

**Table 1:** Different Expression Statistics of RNA-Seq

	Genes	lncRNAs
Total	29,806	17,488
Filter^a^	638	438
> 1.5 Fold change^b^	320	268
< −1.5 Fold change^b^	318	170

^a^Removed FPKM values SD < =15%, fold changes lower than 1.5, or FPKM < 1

If fold change was > 1.5, removed Birc5aMO genes with FPKM less than 1; if fold change was < − 1.5, removed WT genes with FPKM less than 1

^b^Compared average FPKM of Birc5aMO/WT from filtered messenger RNAs (mRNAs) and lncRNAs

Among the identified 438 DE lncRNAs, 408 novel lncRNAs were found in this experiment; the remaining 30 lncRNAs had already been annotated within the zflncRNApedia database. To further analyze the DE lncRNAs and potentially associated genes, we searched for overlapped and nearby genes located within 5 kb of the identified DE lncRNAs; a total of 616 neighboring or overlapped genes, called lncGenes, were retrieved from the zebrafish genome. In biological functional interpretations through GO and KEGG pathway enrichment analysis, several significant GO terms and KEGG pathways were additionally identified using the combination of DE genes and lncGenes (Table 2).

**Table 2:** GO and KEGG enrichment analysis

Annotation	GO^a^	KEGG^a^
Significant items detected from DE genes	165	7
Significant items detected from DE genes + lncGenes^b^	264	28
Additionally detected items	99	21

^a^The results were filtered at *p*-value <= 0.05

^b^Genes associated with DE lncRNAs located at upstream or downstream of the transcription region (within 5 kb)

**Fig. 2:**
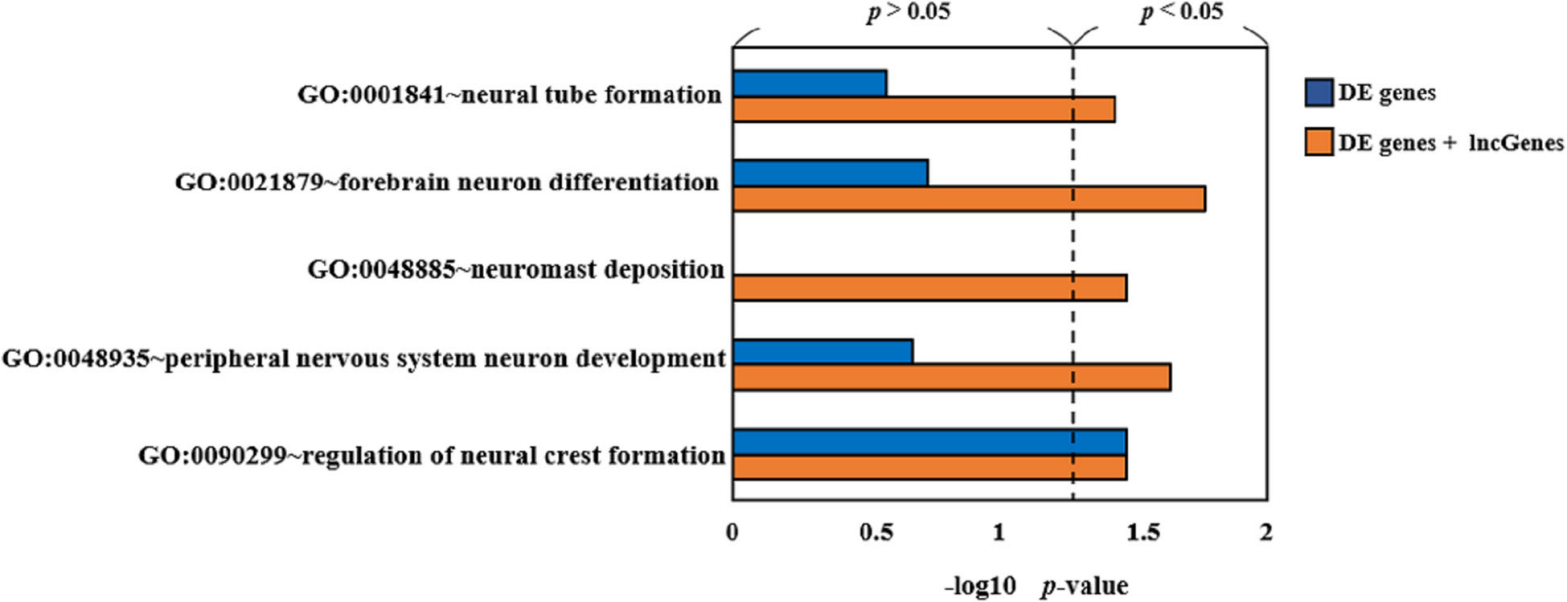
Derived *p*-values of neuron development related GO terms. Blue bars represent using DE genes only and orange bars for using both DE genes and lncGenes for GO enrichment analysis. The significance criterion is *p*-value < 0.05 and located at right half of the plot

When using DE genes and lncGenes, the proposed method detected an additional 99 GO terms compared to the traditional method using DE genes only, of which only 85 GO terms were remained for functional annotations after removing parent GO terms with significant child terms. Similarly, an additional 21 significant KEGG pathways were identified compared to those found by traditional enrichment analysis. In Fig. 2, we selected neuron-related GO terms, which were significantly changed when the additional lncGenes were included in the GO enrichment analysis. When using only DE genes, only one GO terms related to the neuron (GO:0090299) was identified as significant. Comparatively, when additional lncGenes were involved in GO enrichment analysis, four additional GO terms associated with the neuron (GO:0001841, GO:0021879, GO:0048885, and GO:0048935), including neural tube formation, forebrain neuron differentiation, neuromast deposition, and nervous system neuron development, were observed.

**Fig. 3:**
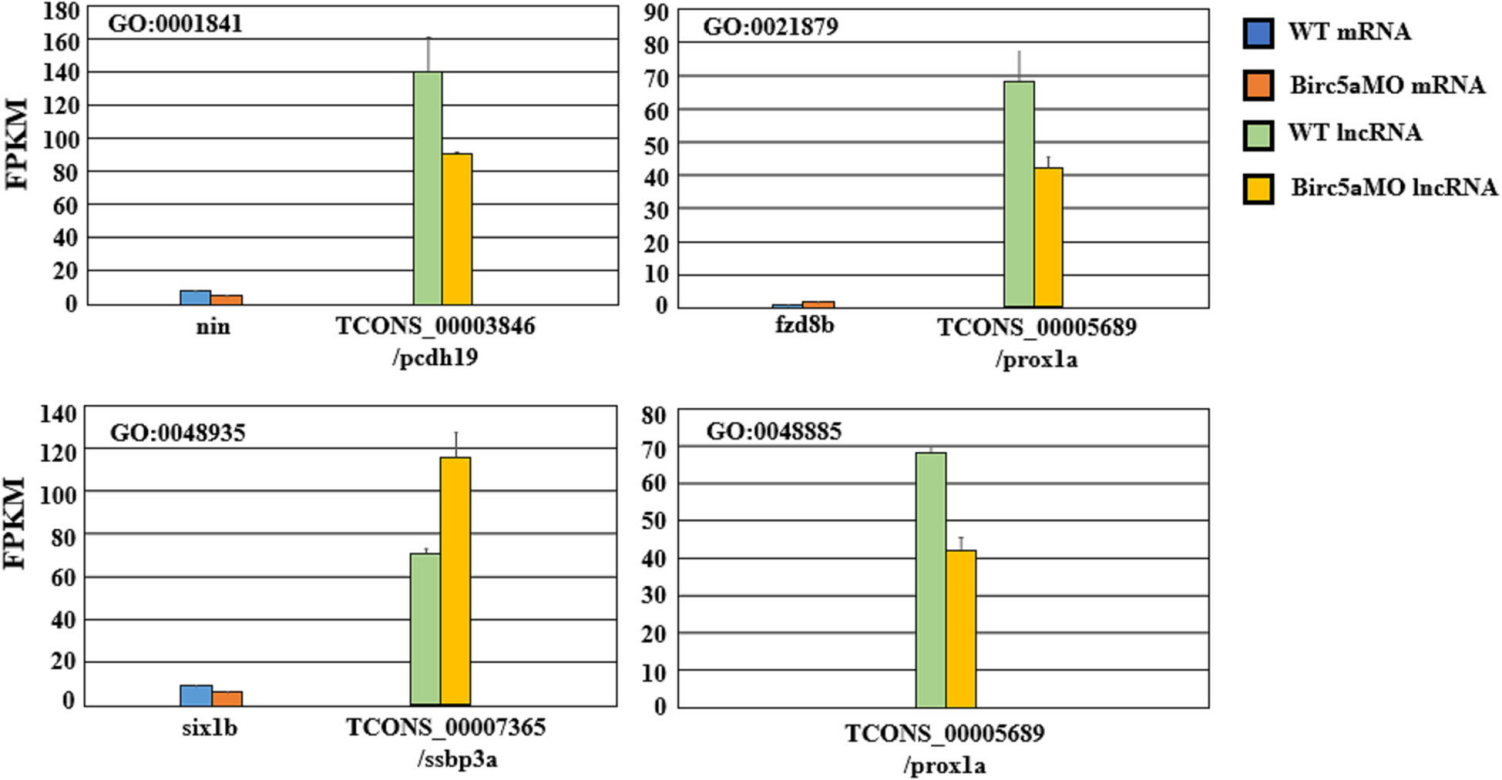
Gene expression values of DE lncRNAs and DE genes related to the additionally identified neuron associated GO terms. FPKM is the measurement of gene expression value. Blue and green bars represent gene and lncRNA expression values in WT dataset respectively; red and yellow bars for Birc5aMO dataset. Genes near or overlapped with the DE lncRNAs were annotated with slash symbols

Figure 3 shows expression values of DE lncRNAs and DE genes annotated within the additionally identified GO terms. These four additional GO terms related to neurons and the brain were retrieved by combing DE genes and lncGenes during enrichment analysis. However, the GO:0048885 term only possessed annotated lncGenes; in other words, this GO term contain no DE genes. All annotated DE genes and DE lncRNAs are shown according to their FPKM expression values for the other four additionally identified GO terms. Notably, FPKM expression values of the DE lncRNAs are relatively greater than those of the genes. These phenomena imply that the identified DE lncRNAs act vigorously and that their associated genes may play important regulatory roles during neuron development. Additionally, all illustrated lncRNAs were novel noncoding RNAs (named with TCONS initials). We also observed that the expression levels of the associated lncGenes did not undergo significant changes in expression. Upon further examination of the distances between the DE lncRNAs and their associated lncGenes, one overlapped with a 5′ exon1 segment (TCONS_00003846), and two overlapped with 3’UTR regions (TCONS_00007365 and TCONS_00005689). Additionally, the lncGene *prox1a* was involved with two additionally detected neuron-related GO terms, which indicates that its DE lncRNA might associate with *prox1a* and play an important role in neuron development through the regulation of *birc5a*.

**Fig. 4:**
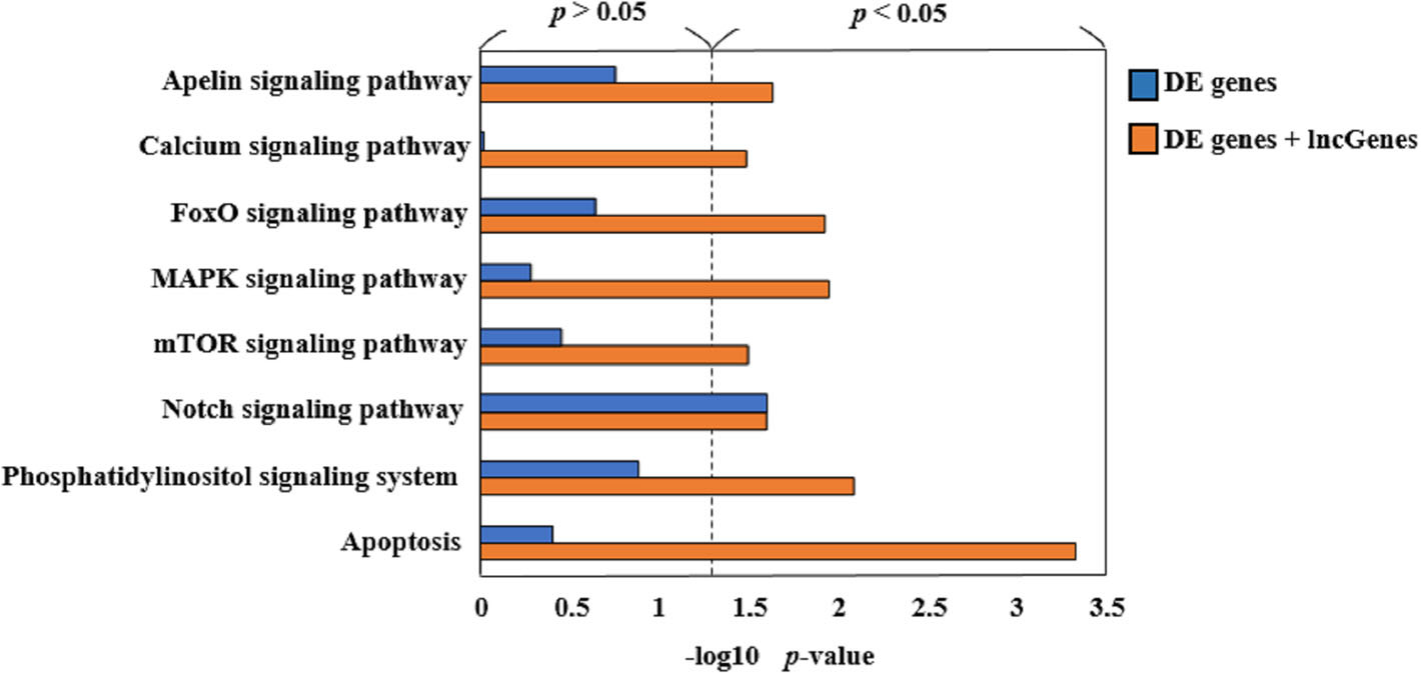
Derived *p*-values for apoptosis-associated KEGG pathways. Corresponding *p*-values are shown in blue and orange bars for traditional and the proposed novel approaches respectively. The significance criterion is *p*-value < 0.05 and located at right half of the plot

**Fig. 5:**
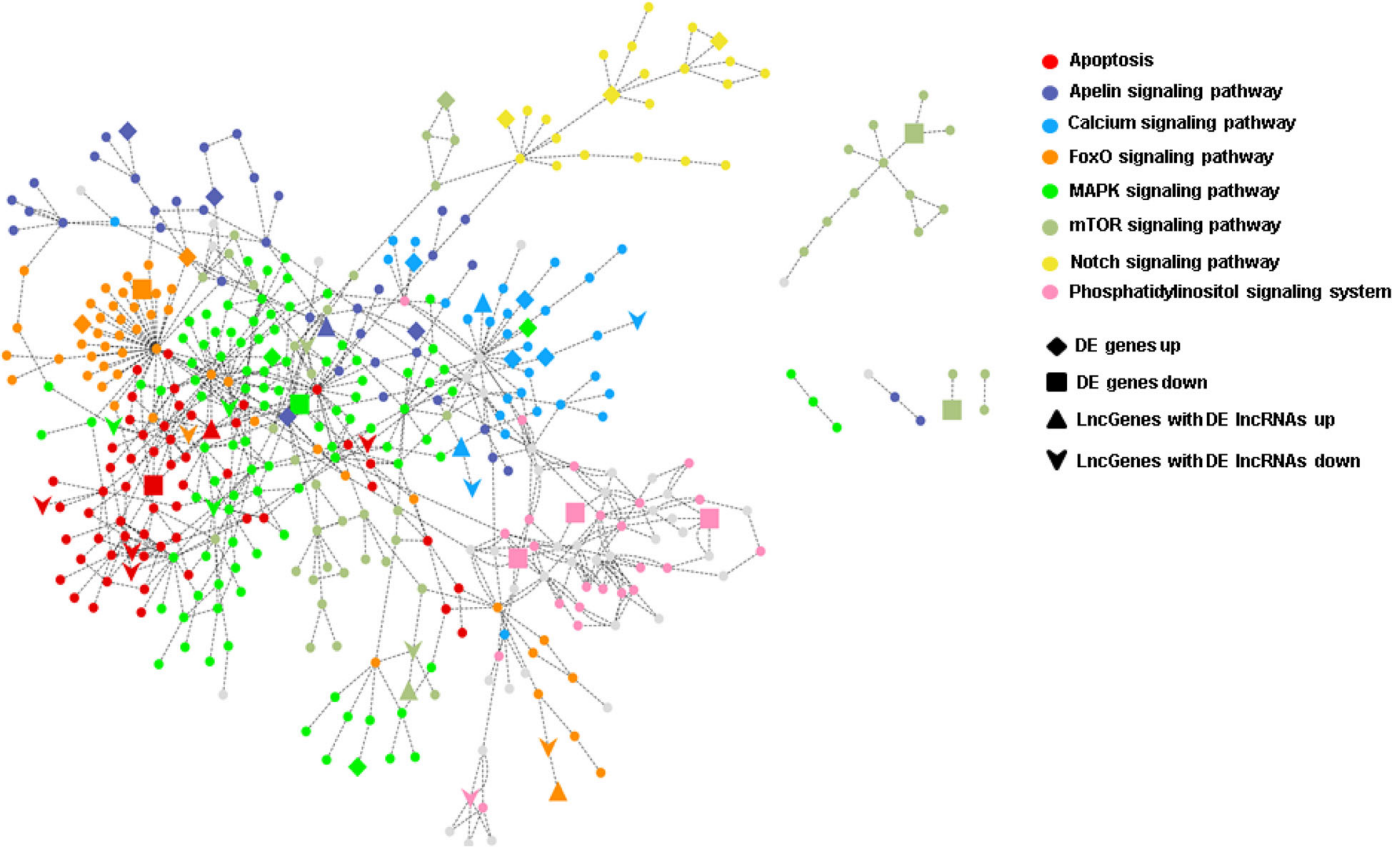
Merged network of apoptosis and seven signal transduction pathways by Cytoscape. FoxO and MAPK signaling pathways are directly connected to apoptosis. MAPK signaling pathway is the central pathway connecting all other pathways. Diamond and square nodes represent DE genes with up- and down-regulated expression respectively. Triangle and arrow shape nodes represent LncGenes (DE lncRNA-associated genes) with up- and down-regulated expression respectively

When using DE genes and lncGenes for KEGG pathway enrichment analysis, a number of additional pathways were found compared to those found using DE genes only. The apoptosis-associated pathways in the survivin gene knock-down experiment are illustrated for comparison along with corresponding *p*-values in Fig. 4. In investigating signal transduction pathways, only the Notch signaling pathway appeared when only DE genes were used in enrichment analysis. Another six signal transduction pathways, including the important MAPK and mTOR pathways, were retrieved by combining DE genes and lncGenes. When integrating the signal transduction and apoptosis pathways, we observed that the FoxO and MAPK signaling pathways were both major sub-networks directly connected to apoptosis (Fig. 5).

Other signal transduction pathways were dispersed around the MAPK pathway, and were not directly connected to the apoptosis cluster. Twelve DE lncRNAs and associated lncGenes were detected among the apoptosis pathway. In the FoxO and MAPK signaling pathways, only two overlapped lncRNAs, *cacna1sboa1* and *lnc2_prkra*, are defined in the zflncRNApedia database. The other novel identified DE lncRNAs were categorized as three lincRNAs (TCONS_00006696, TCONS_00009550, and TCONS_ 00011673), three 3’UTR overlapped lncRNAs (TCONS_ 00004392, TCONS_00009432, and TCONS_ 00012098), one 3′ exon overlapped lncRNA (TCONS_00013138), and three 5’ UTR and/or exon overlapped (TCONS_00008269, TCONS_00010719, and TCONS_00013347). DE genes and lncGenes associated with DE lncRNAs that were simultaneously involved in significant GO terms and KEGG pathways are listed in Table 3. Three DE genes and three lncGenes were detected in the survivin knock-down experiments. Among these identified significant functional terms for both KEGG and GO terms, only the Notch signaling pathway and GO:0007219 (Notch signaling pathway) were identified by traditional approach. All other associated significant pathways and GO terms were identified through our proposed method.

**Table 3:** Overlapped DE genes and lncGenes in KEGG pathways and GO terms

DE gene	FPKM(WT/Birc5aMO)	KEGG^a^	GO^b^
*cacnb4b*	6.88/3.7	MAPK signaling	GO:0070588~calcium ion transmembrane transport
*Dla*	38.61/20.51	Notch signaling	GO:0007219~Notch signaling pathway
*impa1*	8.02/12.31	Phosphatidylinositol signaling system	GO:0046854~phosphatidylinositol phosphorylation
DE lncRNA / lncGene	FPKM(WT/Birc5aMO)	KEGG	GO
*cacna1sboa1* / *cacna1sb*	5.43/2.69	MAPK signaling	GO:0070588~calcium ion transmembrane transport
TCONS_00007367 / *impad1*	99.4/163.82	Phosphatidylinositol signaling system	GO:0046854~phosphatidylinositol phosphorylation
TCONS_00008341 / *tbc1d7*	138.67/208.94	mTOR signaling	GO:0032007~negative regulation of TOR signaling

^a^All pathways are additionally identified except Notch signaling pathway

^b^All GO terms are additionally identified except GO:0007219

## Discussion

In functional analysis of differential expression gene profiles, a group of DE genes clustered and annotated by an identical GO term or a pathway elucidates the responses and effects of transcription changes based on a hypergeometric distribution. Although this approach completely indicates the associations between biological functions and affiliated genes, two major disadvantages exist. First, the significant annotations depend on *p*-value calculations, and if the collected functional annotations are not sufficient, no significant annotations can be identified. Second, most genes affected by ncRNAs are not considered; thus, some pertinent annotations could be ignored due to a statistically insignificant *p*-value. Hence, in traditional DE gene analysis, several hidden gene nodes related to specific biological functions could be neglected. To retrieve the hidden nodes within GO terms and KEGG pathways, ncRNA-associated genes have been proposed to enhance traditional DE gene functional analysis. Our strategy was to consider all transcripts, including mRNAs and ncRNAs, for biological functional annotations. Previous studies reported that lncRNAs were classified into various types: intronic lncRNA, cds overlapping lncRNAs, antisense lncRNAs, and intergenic lncRNAs, among others*.* [21]. However, no single database provides comprehensive annotations for the various types of lncRNAs on regulatory relationships; notably, the lncRNA target gene database (lncRNAs-RNAs) is highly deficient. The adjacent lncRNAs effecting nearby gene expression in fission yeast, *Schizosaccharomyces pombe*, has been discussed. The lncRNA *nc*-*tgp1* increases expression of the neighboring gene *tgp1*^+^ (glycerophosphodiester 1), which is involved in glycerophospholipid metabolism [22]. In female mouse embryonic stem cells, transcription and splicing of the lncRNA Blustr activates Sfmbt2 gene expression and affects protein and histone binding [23]. In their research on E11 mouse embryos, Carlson et al. observed that lncRNA-HIT could enhance developmental transcription factors Hoxa13 and Hoxa11 [24]. Sarangdhar et al. observed a lncRNA called *durga*, which regulates *kalrna* expression to control dendritic cell length and density in zebrafish [25]. LncRNA regulation may be based on different mechanisms, such as co-regulation, Pol ll inhibition, RNA stability, and miRNA regulation [26, 27]. Expression of lncRNAs produced from gene promoter regions probably destabilizes the RNA Pol II transcription-initiation complex, resulting in inhibition of Pol II activity [26]. LncRNAs also regulate mRNA stability; the 3’UTR region of mRNAs may interact with lncRNAs that possess Alu repeat elements, which would induce STAU1-mediated decay (SMD) activities and lead to mRNA decay. Additionally, some lncRNAs such as *bace1as* create RNA-RNA duplexes by combining with mRNAs, and these structure could eliminate miRNA-mediated mRNA instability [28]. The regulatory relationship between lncRNAs and miRNAs can be divided into four categories [29]. First, some lncRNAs are regarded as precursors for miRNA production. Second, miRNAs can bind to lncRNAs directly instead of binding to mRNA. Third, lncRNAs can compete with miRNAs for binding sites on mRNAs. Finally, lncRNAs can act as “sponges” that bind both mRNAs and miRNAs together. LncRNAs can also regulate gene expression by binding to proteins such as transcription factors and help to activate or repress gene expression [30]. Recent studies have shown lncRNAs such as H3K27me3 or H3K4me3 associating with chromatin methylation [13]. Studies have also indicated that lncRNAs bound to proteins involved in chromatin modification, including by methylation of DNA or lysine 9 of histone H3, which control nucleosome structure and result in gene expression changes [31]. LncRNAs bind to chromatin and form protein complexes, and lncRNA-protein complexes interact with methylated chromatin regions to regulate gene expression [32]. Regulation by neighboring lncRNAs has been experimentally demonstrated [23]. Thus, genes overlapped with neighboring DE lncRNAs or within a short range were assumed to be hidden genes participating in functional activities, and the default distance between DE lncRNAs and target genes (lncGenes) was heuristically set at 5 kb as a default value in this study.

To investigate lncRNA expressions and locations in zebrafish, Heena et al. created the lncRNA dataset zflncRNApedia which collected a total of 2267 lncRNAs. However, the dataset only included one RNA-seq datasets from each of the early embryogenesis stage, late developmental stage, and adult tissues. When embryos are stressed under conditions such as hypoxia, some induced or repressed lncRNAs can be observed. Our results indicated that the low expression of *hif2α* in a normoxic environment can invoke Birc5α to protect neural progenitor cells and promote neural differentiation [3]. In this study, we discovered additional neuron-related GO terms using both DE genes and lncGenes (DE lncRNA associated genes) in functional analysis. When using only DE genes to identify significant GO terms, only one neuron-related term was found. We observed that most DE lncRNAs possessed higher FPKM levels than did DE genes among the neuron-related GO terms, which may reflect hidden events regarding the GO functions. When applying DE genes and lncGenes simultaneously, *prox1a* was involved in half of the neuron-related GO terms, which indicated that this gene plays an important role in CNS (central neural system) development. In addition, zebrafish in situ studies have shown that *prox1a* is expressed in the midbrain-forebrain boundary, hindbrain, and spinal cord neurons [33, 34].

For pathway annotations, when using DE genes exclusively in KEGG pathways analysis, only the Notch signaling pathway was detected as significant. However, several signal transduction pathways and apoptosis pathways appeared when using both DE genes and lncGenes (DE lncRNAs associated genes). Survivin is known to play an essential role in apoptosis-mediated neurogenesis [35], and our study observed *birc* gene orthologues possessing the same function as survivin. In addition, the FoxO signaling pathway affiliates multiple biological functions, including apoptosis, glucose metabolism, and oxidative stress response. Activation of FOXO3/FKHRL (key genes in FoxO signaling pathway) would induce repression of *birc5*/*survivin* and apoptosis of neuroblastoma cells [36]. Pathway analysis showed FoxO and MAPK were the main pathways connected to apoptotic processes. Three pathways, including MAP/ERK, JNK/p38, and ERK5, belonged to the subnetworks of the MAPK pathway. The DE genes and lncGenes showed that regulation of the MAP/ERK and JNK/p38 pathways is related to cell proliferation and apoptosis. Previous studies have indicated that the MAP/ERK signaling pathway is necessary for CNS development [37], and that the JNK/p38 signaling pathway plays an essential role in the differentiation of neural stem cells [38].

The other signaling transduction pathways located around the MAPK pathway are not connected to apoptosis directly, but their functions are also crucially related to neuron development. Several Notch signaling pathway studies on neuronal development and neuronal degeneration have been reported [39]. The phosphatidylinositol signaling pathway is important in intra- and extra-cellular signaling and affects neuron development [40]. TOR signaling has also been reported as a key molecular pathway in nervous system development [41]. The calcium signaling pathway is a neuronal-specific signaling pathway [42], and several DE genes and lncGenes annotated in the MAPK/apoptosis pathways are also annotated in GO functional analysis of the calcium signaling pathway. Many studies have observed the effects of calcium on neurons; Duchen reported that neuronal cell death was mediated by calcium signaling [43]. Turner reported that calcium-activated potassium channels expressed in excitatory and inhibitory cells played a role in CNS development [44]. Calcium influxes into neurons increases ATP production, which stimulates the citric acid cycle in mitochondria, supplying energy for neuronal metabolism [45]. These described pathways were identified by using both DE genes and DE lncRNA-associated genes (lncGenes) in KEGG pathway enrichment analysis.

## Conclusions

We have proposed a method that combines expression changes of genes and lncRNAs for biological functional analysis. Our results suggest that ncRNAs play essential roles in regulating gene expression. The retrieval of additional significant GO terms and KEGG pathways provided important context for understanding the regulation of *birc5a* in embryonic neuron development. Further research verifying the target genes of associated DE lncRNAs will be conducted through biological experiments for clarification.

## Abbreviations

Birc5aMO: Birc5a morpholino-treated
DE lncRNA: Differentially expressed long noncoding RNA
DE: Differential expression
DEG: Differentially expressed gene
FPKM: Fragments Per Kilobase of transcript per Million
GO: Gene ontology
lncGene: lncRNA-associated gene
lncRNA: Long noncoding RNA
miRNA: Microrna
mRNA: Messenger RNA
ncRNA: Noncoding RNA
WT: Wild type
